# Diffuse Large B-Cell Lymphoma in the HIV Setting

**DOI:** 10.3390/cancers15123191

**Published:** 2023-06-15

**Authors:** Maria Huguet, José-Tomás Navarro, José Moltó, Josep-Maria Ribera, Gustavo Tapia

**Affiliations:** 1 Department of Hematology, Institut Català d’Oncologia, Germans Trias i Pujol University Hospital, Universitat Autònoma de Barcelona, Ctra. de Canyet, S/N, 08916 Badalona, Spain; mhuguetm@iconcologia.net (M.H.); jribera@iconcologia.net (J.-M.R.); 2 Josep Carreras Leukaemia Research Institute, 08916 Badalona, Spain; 3 Fundació Lluita Contra les Infeccions, Infectious Diseases Department, Germans Trias i Pujol University Hospital, Ctra. de Canyet, S/N, 08916 Badalona, Spain; jmolto@lluita.org; 4 Centro de Investigación Biomédica en Red de Enfermedades Infecciosas (CIBERINFEC), 28029 Madrid, Spain; 5 Department of Pathology, Germans Trias i Pujol University Hospital, Universitat Autònoma de Barcelona, Ctra. de Canyet, S/N, 08916 Badalona, Spain; gtapia.germanstrias@gencat.cat

**Keywords:** HIV, diffuse large B-cell lymphoma, antiretroviral therapy, prognosis

## Abstract

**Simple Summary:**

Non-Hodgkin lymphoma (NHL) is one of the most frequent HIV-related neoplasms, and diffuse large B-cell lymphoma (DLBCL) is the most common subtype. In people with HIV (PWH), DLBCL classically presents with aggressive characteristics. As in the general population, HIV-related DLBCL is a heterogeneous disease that includes morphological and molecular subtypes. When combined antiretroviral therapy (cART) became widely available, a strong improvement of the immune function and a better management of infectious complications during lymphoma treatment were observed in PWH with lymphoma. Moreover, the concomitant treatment of cART with chemotherapy was shown to be beneficial. These changes have led to a marked improvement in prognosis for PWH with DLBCL, approaching that of the general population.

**Abstract:**

Despite the widespread use of combined antiretroviral therapy (cART) and the subsequent decrease in AIDS-defining cancers, HIV-related lymphomas remain a leading cause of morbidity and mortality in people with HIV (PWH). Diffuse large B-cell lymphoma (DLBCL) is the most common non-Hodgkin lymphoma (NHL) subtype in PWH. This lymphoma is a heterogeneous disease including morphological variants and molecular subtypes according to the cell of origin or the mutation profile. In the pre-cART era, treatment with standard-dose chemotherapy induced high rates of toxicity and outcomes were very poor. The introduction of cART and the incorporation of infection prophylaxis allowed the use of conventional intensive chemotherapy regimens used in the general population, such as R-CHOP or R-EPOCH. The use of cART during chemotherapy treatment was initially controversial due to the potential risk of adverse drug–drug interactions. However, the availability of current cART regimens with less potential to cause drug interactions and evidence that cART improves survival rates in NHL strongly support the use of cART in PWH with DLBCL. Consequently, interdisciplinary collaboration between HIV specialists and hemato-oncologists for the management of potential interactions and overlapping toxicities between antiretroviral and antineoplastic drugs is crucial for the optimal treatment of PWH with NHL.

## 1. Introduction

HIV infection has a direct impact on the development of some cancers due to the effect of the HIV on CD4^+^ T-cells and the impaired immunosurveillance [1]. Moreover, people with HIV (PWH) frequently present viral co-infections, such as Epstein–Barr virus (EBV) and human herpesvirus-8 (HHV8), which are known to be involved in lymphomagenesis [2]. In the early years of the AIDS pandemic, the association of HIV infection with several hematological malignancies was included in the 1993 US Centers for Disease Control and Prevention AIDS definition [3], including diffuse large B-cell lymphoma (DLBCL), Burkitt lymphoma (BL), and primary central nervous system lymphoma (PCNSL) [4]. Before the development of effective combined antiretroviral therapy (cART), the relative risk of NHL was estimated at 60 to 200-fold, compared with the general population and, in particular, 98-fold for DLBCL [4]. 

The widespread use of cART led to a substantial improvement in life expectancy for PWH. This resulted in changes in the demographics of the population of PWH, who are nowadays older, mostly virologically suppressed, and generally have higher CD4^+^ T-cell counts [5]. In this context, the incidence of AIDS-related cancers in PWH has decreased [6], but cancer incidence remains higher in PWH than among the general population [7]. Currently, NHL is the most frequent HIV-related neoplasm in developed countries, and it is still one of the most frequent neoplastic causes of death among PWH [8]. Although its incidence declined after the introduction of cART, DLBCL is still the most common subtype of NHL occurring in this population [9,10]. 

In the pre-cART era, treatment of aggressive lymphomas with standard-dose chemotherapy was associated with high rates of toxicity and opportunistic infections [11]. Low-dose chemotherapy and risk-adapted intensive chemotherapy regimens were evaluated, with poor outcomes [12]. After the introduction of cART, patients with HIV-related NHL presented enhanced immunity, better functional status, and higher tolerability to standard chemotherapy [10]. Therefore, patients with HIV-related DLBCL are currently treated with the same regimens as those given to the general population, achieving similar response rates [13,14,15,16]. 

Although the cART and the improvements in antineoplastic treatment have produced prolonged survival in PWH and lymphoma, the survival of those with NHL, including DLBCL, is still different in PWH than in the general population [17], perhaps because of the increasing incidence of non-AIDS defining cancers and HIV-comorbidities, both related to the longer survival of PWH. In this article, the authors review and update the epidemiological, clinical, and biological aspects of DLBCL presenting in PWH, with special emphasis on the changes in epidemiology, treatment, and prognosis over the past few decades. 

## 2. Epidemiology

In 2021, an estimated 38.4 (33.9–43.8) million people lived with HIV infection worldwide, of whom three quarters were receiving cART [18]. Overall, PWH have an increased risk of hematologic cancers [19,20,21]. B-cell aggressive NHL was included in the 1985 revised case definition of AIDS as one of three AIDS-defining cancer categories [3] and, currently, it is the most common hematological malignancy in PWH [22,23,24,25]. Nowadays, the most frequent types of lymphoma are DLBCL, decreasing from 63% in the pre-cART era (1986–1995) to 35–37% in the late-cART era (2006–2015), and BL, increasing from 3% in the pre-cART era to 16–20% in the late-cART era [17]. As shown in the large cohort study of the CNICS USA (Table 1), since the introduction of cART, the incidence of PCNSL and systemic DLBCL (specially the immunoblastic variant) has decreased. In contrast, the burden of HIV-related BL and Hodgkin lymphoma has increased [5]. Primary effusion lymphoma (PEL) and plasmablastic lymphoma (PBL) occur nearly exclusively in PWH [6], and their incidence has remained stable through the decades [5]. NHL occurring in PWH is closely linked to other viral infections: DLBCL, BL, and PBL are associated with EBV infection [26], and PEL is linked with HHV8 infection [27]. 

In the general population, the estimated lifetime risk of NHL is 1 in 108 for men and 1 in 162 for women [28]. These kinds of data for the global community of PWH are not available, but it is known that HIV infection increases the risk of NHL. A meta-analysis of population-based studies of cancer risk among PWH showed a standardized incidence ratio of 76.7 for NHL [29]. Another study in North America estimated a lifetime risk of about 1 in 25 [7]. Retrospective specific epidemiology data about DLBCL in PWH are hard to find nowadays, due to most of the studies reporting heterogeneous data about NHL or lymphoid neoplasms in general. 

In the mid-1990s, developed countries performed HIV-treatment programs, and the first studies and registries of cancer in PWH were rolled out, showing heterogeneity among NHL subtypes [6]. In the USA, from 1996 to 2010, the incidence of NHL was 193 per 100,000 person-years, increasing 11 times more in PWH than in the general population [30]. Similar increases (about 10 to 20 times) were reported from other countries, such as Germany, France, Sweden, and Italy [30,31,32,33]. Regarding developing countries, the incidence of NHL for PWH is low compared with rates seen in Europe and the USA [6]. For example, a study performed in South Africa from 2004 to 2010 estimated an incidence rate of 85/100,000 person-years [34]. Similarly, in a study performed in Uganda, an incidence rate of 19/100,000 person-years was found [35], which is much lower than in the USA (193/100,000 person-years) over a similar period [30]. The lower reported incidence of NHL in the African studies is probably related to the underdiagnosis and the increased mortality from other competing causes, such as opportunistic infections [6]. 

HIV-related DLBCL is more common in men than in women, which is consistent with the sex ratio in the general population [36]. HIV-related NHL is generally a late event during HIV infection and risk factors for its development include a low CD4^+^ T-cell count and high HIV viral load [36]. Among B-cell NHL, PCNSL is associated with greater immunosuppression and presents with the lowest CD4^+^ T-cell count at diagnosis [5]. Both profound immunosuppression and prolonged viremia greatly increase the risk of DLBCL [37].

The development of new and more robust antiretroviral drugs and the improved access to cART within the last decades have resulted in a higher proportion of patients with undetectable viral load and higher CD4^+^ T-cell counts [6]. In this context, the incidence of PCNSL and DLBCL have decreased [38]. The introduction of modern cART has also delayed the age of patients at DLBCL diagnosis, although it continues to occur a decade earlier than in the general population, mostly during their fifth decade of life [5,31].

## 3. Pathological Characteristics and Etiopathogenesis

HIV-related DLBCL is histologically indistinguishable from DLBCL in the general population. However, some cases may show Hodgkin or Reed–Sternberg-like cells, raising the differential diagnosis with Hodgkin lymphoma (HL) or with polymorphic lymphoproliferative disorder. There are two main different morphological variants of DLBCL: the centroblastic variant (CB), composed of centroblasts with multiple nucleoli, and the immunoblastic variant (IB), composed of immunoblasts with a single and prominent nucleolus (Table 2) [12,17]. The immunoblastic variant is considered to occur with more frequency in patients with more advanced HIV disease when compared with centroblastic variant [39]. Moreover, it is known that HIV-related primary DLBCL of the CNS often presents with the immunoblastic type [12]. In HIV-related DLBCL, there are more frequent plasmacytoid features compared to the general population [39]. Although these cells present features of the immunoblastic stage of B-cell development, they also display a plasma-cell-related phenotype, expressing plasma cell surface markers, such as CD138, while mature B-cell markers (CD20, CD45) are often downregulated [27]. The prognostic significance of the immunophenotypic characteristics is still unclear. 

In the early 2000s, gene-expression profiling studies of DLBCL performed in the general population identified two transcriptional subgroups considering the supposed cell of origin (COO): germinal center B-cell subtype and activated B-cell subtype [39]. The germinal center B-cell subtype of DLBCL corresponds to B-cells that are arrested at various stages of the germinal center transits, and the activated B-cell subtype of DLBCL seems to derive from germinal center B-cells evolving through plasma cell differentiation [17,39]. These DLBCL subgroups have different prognoses and may have relevance for treatment [39]. However, the predictive power of the molecular classification is uncertain, and some data suggested that it is not clinically relevant for prognosis in HIV-related DLBCL [40]. In contrast, most recent studies pointed out that HIV-related DLBCL cases with a non-germinal center phenotype tended to have a worse overall survival and progression-free survival than the cases with the germinal center phenotype [41,42].

Most of the evidence suggests that the etiopathogenesis of HIV-related NHL is a progressive multistep process, which involves viral and host factors and specific changes in the tumor clone [43]. Human immunodeficiency virus is not known to have direct oncogenic effects, but HIV infection causes several indirect effects, which are implicated in lymphomagenesis. Recent evidence suggests that HIV may contribute to lymphomagenesis by acting directly on B-cells, as a critical microenvironmental modifier [1]. In a systemic background of immunodeficiency (minimized by cART), B-cells receive an uncontrolled chronic activation through persistent antigenic stimulation, HIV CD40 ligand (CD40L), and HIV-encoded proteins, such as gp120, p17, and TAT [1,44]. Moreover, HIV infection leads to a loss of immune surveillance because of depletion of T lymphocytes, and reactivated oncogenic viral infection, such as Epstein–Barr virus (EBV), human herpesvirus 8 (HHV8), and chronic antigen stimulation mediated by other viral co-infections, including hepatitis B and C viruses [6,37]. In this way, co-infection with EBV or HHV8 contributes to certain subtypes of aggressive B-cell non-Hodgkin lymphoma development. Regarding DLBCL, EBV infection occurs in a proportion of cases, being more frequent in IB variants [17]. HHV8 is causally associated with primary effusion lymphoma and, in fact, HHV8 infection of the cells is a diagnostic requirement [45]. Aberrant B-cell activation and/or EBV infection may upregulate CXCR2 and IL-8 receptor, which are cellular receptors for p17 variants. CXCR2 upregulation by different p17 variants promotes B-cell expansions, increasing the probability of acquiring critical genetic alterations (involving *MYC*, *BCL6* mutations, and other molecular events.) [1]. Moreover, in EBV-infected B-cells, p17 variants may upregulate LMP-1, the main EBV carcinogenic protein, which would contribute to lymphomagenesis [1]. 

EBV infection occurs in 90–100% of the cases with IB morphology and 25–30% of the DLBCL cases with CB morphology [1,17,44]. In general, positivity for EBV is found in about 31% of the HIV-related DLBCL cases [46]. After the primary infection, EBV spreads throughout lymphoid tissues and infects B-cells. Infected B-cells develop a primary cytotoxic T-cell response that would control the EBV infection and establish a reservoir of memory B-cells with latent viral expression. There are three known latency patterns (type I, II, and III) [2]. As suggested in previous studies, EBV seems to be involved in different pathways depending on the DLBCL cell of origin subtype. Arvey et al. reported that 76% of germinal center B-cell cases are associated with latency type I (LMP1−, EBNA2−), 12% with latency type II (LMP1+, EBNA2−), and 12% with latency type III (LMP1+, EBNA2+). On the other hand, in the activated B-cell subtype, types II or III latency were both observed in 30% of the cases, and latency type I in 37% of the cases [44].

EBV-positive HIV-related DLBCL presents a high expression of BLIMP1, repressing p53 transcription, conferring the ability to avoid apoptosis [46]. Furthermore, HIV-related DLBCL with EBV infection is associated with CD30 expression [46]. CD30 stimulates the activation of the NF-κB pathway, which is associated with cellular proliferation and carcinogenesis [46]. Moreover, other genetic alterations involving *MYC*, *BCL6*, and *TP53* genes are frequently identified in HIV-related DLBCL [2]. Additionally, aberrant somatic hypermutations involving *PIM1*, *PAX5*, and *RhoH*/*TTF* have been reported in about 50% of the cases [2].

Due to the genetic and phenotypic heterogeneity of DLBCL, a DLBCL taxonomy was defined with regard to the genetic pattern. Schmitz et al. [47] identified four prominent genetic subtypes in DLBCL, termed MCD (co-occurrence of MYD88L265P and CD79B mutations), BN2 (*BCL6* fusions and *NOTCH2* mutations), N1 (*NOTCH1* mutations), and EZB (*EZH2* mutations and *BCL2* translocations). Similarly, Chapuy et al. grouped DLBCL cases in five clusters according to their molecular characteristics [48]. Recently, Wright et al. [49] developed the LymphGen Classifier, unifying the two previous studies, and Lacy et al. [50] also confirmed the existence of reproducible molecular subtypes of DLCBL defined by their profile genomic alterations, detected using a targeted sequencing panel applied to biopsy material. These classifications break DLBCL into different genetic subtypes that differ with respect to oncogenic pathway, gene-expression phenotype, tumor microenvironment, survival rates, and potential therapeutic targets [49]. These studies have not been validated in HIV-associated DLBCL cohorts. 

It is known that the tumor microenvironment and its interaction with viral components in HIV-related DLBCL play a crucial role in lymphomagenesis. HIV-related DLBCL is highly angiogenic and shows a markedly higher blood-vessel density than sporadic DLBCL cases [51]. Some studies suggest that EBV infection may be related with these angiogenic properties [51]. The duration of immunodeficiency, measured as the time since HIV-seroconversion, and the degree of chronic B-cell stimulation also have a role in lymphomagenesis and are measured mainly by means of two markers: raised serum immunoglobulin concentration and HIV p24 antigenemia [43].

## 4. Clinical Characteristics, Treatment, and Prognosis

Regarding the clinical features, HIV-related DLBCL is characterized by advanced-stage disease at diagnosis [36], the presence of B symptoms with more frequency than in the general population, and extranodal involvement at diagnosis, including bone marrow infiltration [52] and leptomeningeal disease [53]. Some studies performed in the cART era suggest that CNS involvement would have decreased, currently with a frequency similar to that of the general population [54,55]. Unusual extranodal locations are often reported, such as oral cavity, adrenal glands, kidney, lung, or bladder [36]. As described in the Center for AIDS Research Network of Integrated Clinical Systems cohort of patients with HIV-related lymphoma between 1996 and 2010, the median CD4^+^ T-cell count at diagnosis of HIV-related DLBCL is about 120/μL [5].

Lymphoma may be the presenting manifestation of HIV infection, and all patients with aggressive B-cell lymphoma should be tested for HIV [56]. Diagnosis of lymphoma should be based on an excision biopsy [11]. Before starting the treatment, a staging procedure, including the same tests as the general population, should be performed. A basal ^18^Fluoro-2-deoxy-D-glucose positron emission tomography (^18^F-FDG-PET) scanning at diagnosis improves the staging accuracy [11,57]. Trephine biopsy should be performed to rule out bone marrow involvement [11]. HIV-related NHL is characterized by advanced-stage disease with an aggressive clinical course, and CNS involvement in HIV-related NHL occurs in about 13–20% of the cases [55]; so, for most patients with HIV-related DLBCL, staging should also include evaluation for CNS involvement, with cytologic and flow cytometric analysis of the cerebrospinal fluid [56,58].

### 4.1. First-Line Treatment

In the pre-cART era, treatment with standard-dose chemotherapy induced high rates of toxicity and response rates were counterpoised by increased death due to opportunistic infections [11]. In this context, low-dose chemotherapy and risk-adapted intensive chemotherapy regimens were administered to PWH [12]. Outcomes were poor, with complete remission rates of about 50% and 5-year overall survival of about 28–47%, which was by far inferior to that observed in patients without HIV infection [59,60,61,62].

The introduction of cART led to an improved immune function in HIV patients, and the incorporation of infection prophylaxis and hematopoietic growth factors into treatment protocols allowed an increased use of conventional standard-dose chemotherapy regimens [11]. The first-line treatments in PWH were the same used for the general population: CHOP (cyclophosphamide, doxorubicin, vincristine, and prednisolone) [55,56] or infusional regimen, dose-adjusted (DA) EPOCH (etoposide, prednisone, vincristine, cyclophosphamide, and hydroxydaunorubicin) [41,63,64]. In some centers, DA-EPOCH was preferred over CHOP, due to superior response rates observed when compared to historical data [64,65,66]. However, a randomized study comparing CHOP vs. EPOCH in HIV patients has not been performed. Other centers have reported experience with infusional regimen of CDE (cyclophosphamide, doxorubicin, and etoposide) [67], but there are no available data comparing CDE to CHOP. Guideline recommendations for HIV-related DLCBL are in the same line as those for DLCBL in HIV-negative patients, and CHOP is considered the standard first-line therapy [68,69,70]. 

The addition of the anti-CD20 monoclonal antibody rituximab to standard chemotherapy regimens for B-cell NHL treatment has demonstrated a significant survival benefit in the general population. However, the use of rituximab in HIV-related DLBCL has been controversial [71,72]. In 2003, an AIDS Malignancy Consortium randomized phase 3 study with CHOP alone vs. CHOP with rituximab (*n* = 150) was performed, and a trend was found for better outcomes associated with the use of rituximab [73], but an increased frequency of infectious-related deaths in the rituximab group was detected (14% vs. 2%, *p* < 0.001), particularly in those patients with CD4^+^ lymphocyte counts <50/μL [73]. Subsequent studies demonstrated that the combination of R-CHOP, R-CDE, R-EPOCH, or DA-EPOCH-R was beneficial (Table 3), with an improvement in complete response rate (69–91%) [41,64,71,74,75,76], in 2-year progression-free survival (59–69%) [64,71,74] and in 2-year overall survival rate (64–75%) [58,65,68], with a lower infectious death rate (<10%) [41,64,71,74,75,76]. In a pooled analysis of 1546 patients, rituximab was associated with higher CR rate (odd ratio, 2.89; *p* < 0.001), improved progression-free survival (PFS; hazard ratio 0.50; *p* < 0.001), and OS (hazard ratio, 0.51; *p* < 0.0001) [77]. 

Despite this evidence, the use of rituximab in patients with CD4^+^ lymphocyte counts <50/μL is still controversial [72]. In the cART era, treatment outcomes significantly improved for the patients with HIV-related NHL with CD4^+^ lymphocyte counts <50/μL, and the 2-year OS increased to 65% from 16% in the pre-cART era [41]. In this way, the observational study from Wyen et al. showed no association between rituximab use and infectious death risk in patients with a CD4^+^ count lower than 100/μL [79]. Nowadays, there is a consensus for rituximab use in all patients with HIV-related CD20-positive NHL, with special care in infectious prophylaxis in high-risk patients [12,17].

Several groups have pointed out that CDE or EPOCH infusional chemotherapy regimens [41,64] are associated with less tumor resistance, less cardiac toxicity, and the addition of a synergic effect with the rituximab combination [80], and could be a better option in patients with HIV-related NHL. Analysis of the results of different trials from the AIDS Malignancy Consortium has suggested that in patients with HIV-related B-cell NHL, there is a higher efficacy for infusional R-EPOCH compared to R-CHOP bolus [65]. Improved event-free survival (EFS; hazard ratio, 0.4; *p* < 0.001) and overall survival (OS; hazard ratio, 0.38; *p* < 0.01) were observed, and this difference was especially remarkable in patients with high-risk IPI [65]. However, a randomized trial in immunocompetent patients with DLBCL showed that DA-EPOCH-R and R-CHOP were equally effective but with greater toxicity and complexity of the infusional therapy [81]. It is controversial whether or not patients with a high-risk IPI could benefit from DA-EPOCH-R [81]. 

### 4.2. Relapse/Refractory Lymphoma

High-dose chemotherapy platinum-based salvage regimens (e.g., R-DHAP, R-ICE, R-GDP, and R-EHAP) and subsequent autologous stem-cell transplantations (ASCTs) are strategies that have been used in relapsed/refractory HIV-related lymphoma [82,83,84]. A multicentric study demonstrated that HIV infection has no impact on the long-term outcome of ASCT for lymphomas [85]. The largest prospective study of ASCT in PWH was reported by the GICAT (Italian Cooperative Group on AIDS and Tumors), with 50 patients with relapsed/refractory HIV-related lymphoma, achieving a PFS and OS after 4 years of follow-up, of 75% and 76%, respectively, among the 27 patients who had undergone ASCT [82]. Regarding the role of allogeneic hematopoietic stem-cell transplantation (HSCT), recent studies suggest that in the cART era it could be a feasible option for PWH with high-risk relapsed malignancies. However, interactions between immunosuppressive drugs and antiretroviral agents, the presence of some degree of graft-versus-host-disease, and the high incidence of infectious complications should be taken into account [86]. Selected PWH with hematologic malignancies should be considered for allogeneic HSCT when indicated, in experienced centers [86]. A few case reports have shown the feasibility of manufacturing CD19 CAR-T-cells in PWH and a successful outcome in some cases [87,88]. 

Unfortunately, most clinical trials for new agents and for cell therapy still exclude PWH [89], and there is limited evidence for novel agents, such as polatuzumab vedotin, brentuximab vedotin, lenalidomide, and proteasome inhibitors in the HIV setting.

### 4.3. Antiretroviral Therapy during Chemotherapy

Maintenance or initiation of cART concomitantly with chemotherapy has demonstrated an improved complete response rate [77,90,91,92,93] and immune recovery after chemotherapy [94]. The risk of drug interactions associated with the use of strong cytochrome 3A4 inhibitors such as ritonavir- or cobicistat-based regimens has been overcome with new antiretroviral agents with fewer drug interactions, such as unboosted integrase strand-transfer inhibitors (INSTIs) [17]. Therefore, most current guidelines recommend the use of cART during chemotherapy in PWH and lymphoma [77,95], preferably with unboosted INSTIs [17]. 

Clinically significant interactions between chemotherapy regimens and cART have been reported, with a higher risk with regimens with ritonavir or cobicistat (boosters). These two agents are potent inhibitors of cytochrome P450 enzymes and could change the disposition of numerous drugs, leading to marked increases in drug exposure [96,97]. Of note, the use of boosters in PWH with lymphoma has been associated with higher probability of dose-reduction as well as with worse overall survival [98,99]. Similarly, the use of ritonavir increased the risk of adverse events in PWH receiving CHOP [100,101]. In addition, an increased autonomic neurotoxicity in patients receiving lopinavir/ritonavir with vincristine was described by Leveque et al. [102]. 

Conversely to ritonavir or cobicistat, some non-nucleoside reverse transcriptase inhibitors (NNRTIs) such as nevirapine, efavirenz, and etravirine are moderate to potent cytochrome P450 inducers. Consequently, their use in PWH and lymphoma could potentially reduce the exposure, and thus the efficacy, of certain chemotherapy drugs [103]. Rilpivirine and doravirine are second-generation NNRTIs that do not induce the P450 system, limiting their potential for interactions with chemotherapy [104,105]. As previously mentioned, cART with unboosted INSTIs may be particularly recommended for PWH and lymphoma due to their favorable efficacy, safety, and drug interactions profile. Raltegravir, dolutegravir, or bictegravir do not exert any inducer or inhibitory effect on P450 enzymes or drug transporters, minimizing their potential for drug interactions [106,107,108]. 

The HIV capsid inhibitor lenacapavir and the HIV attachment inhibitor fostemsavir are two new drugs recently approved for the treatment of HIV infection. Although these drugs do not need to be boosted by ritonavir or cobicistat, they may exert some interactions that need to be considered. Lenacapavir is a moderate inhibitor of the isoenzyme CYP3A4 of the P450 system, and it may increase the exposure to other drugs that are metabolized by CYP3A4. Consequently, caution is advised when lenacapavir is co-administered with sensitive CYP3A4 substrates with a narrow therapeutic index [109]. Similarly, temsavir (the active moiety of fostemsavir) inhibited the drug transporters OATP1B1/3 and BCRP in vitro, and administration of fostemsavir is expected to affect the pharmacokinetics of active substances that are substrates of OATP1B1/3 or BCRP. Therefore, close monitoring is recommended, and eventual dose modifications may be needed [110]. 

Importantly, antiretroviral agents may also interact with other common drugs, such as omeprazole or other proton pump inhibitors, which reduces the bioavailability of rilpivirine, resulting in a decrease in the antiviral action [104]. In the same way, antiacids containing divalent cations can reduce INTIs absorption [111,112]. An antiretroviral regimen can be changed before starting a chemotherapy regimen to avoid drug interactions and reduce toxicity in order to improve tolerability and adherence. Discontinuation of regimens with ritonavir or cobicistat is recommended, but the discontinuation of a single drug from the antiretroviral regimen must be avoided, because it may decrease the efficacy of cART and favors viral resistance. Modifications in cART regimens should be consulted with an HIV specialist and interdisciplinary evaluation is mandatory in order to decide the best treatment for both the hematologic malignancy and the HIV infection [113]. 

### 4.4. Additional Measures and Supportive Care

Infection prophylaxis must be performed in PWH receiving cancer treatment [11,12,114,115]. *Pneumocystis jiroveci* prophylaxis is indicated in PWH receiving chemoradiotherapy because chemotherapy can markedly reduce CD4^+^ T-cell counts, even in patients on cART. *P. jiroveci* prophylaxis is usually performed with oral cotrimoxazole three times per week (800 mg trimethoprim and 160 mg sulfamethoxazole). Aerosolized pentamidine 300 mg once a month, dapsone 100 mg daily, or atovaquone 1500 mg daily are alternative options. Prophylaxis for *Mycobacterium avium complex* should be considered for patients with CD4^+^ counts <50–100/μL, with oral azithromycin 1200–1250 mg per week or rifabutin 300 mg daily. Primary prophylaxis of the chemotherapy-related neutropenia with a granulocyte colony-stimulating factor must be performed, starting 48–72 h after chemotherapy. In patients with neutrophil count of <100/μL or neutropenia lasting more than 7 days, antibacterial prophylaxis with levofloxacin could be considered, although it could favor the emergence of multiresistant bacteria. PWH with low CD4^+^ T-cell count are at an increased risk of fungal infections, particularly candidiasis and cryptococcosis. Therefore, fluconazole is recommended for PWH receiving chemotherapy. PWH treated with intensive chemotherapy should receive acyclovir or valacyclovir for herpes simplex and varicella prophylaxis. Monitoring for cytomegalovirus infection must be performed. Patients with hepatitis B virus infection or positive anti-HBc receiving chemotherapy should be treated with antihepatitis B virus agents. Patients with hepatitis C virus infection must receive antiviral treatment even if the fibrosis stage is detected [116,117]. Moreover, all PWH with hematological malignancies should receive annual vaccination against SARS-CoV-2 and influenza [116,117,118].

### 4.5. Prognosis

During the pre-cART era, HIV-related NHL outcomes depended specially on HIV-related factors, such as a poor bone marrow reserve, CD4^+^-cell count <100/μL, prior AIDS-defining illness, HIV viral load, or the development of an opportunistic infection [36]. In the Straus et al. study, an index was developed which included CD4^+^-cell count, age higher than 35 years, stage III or IV disease, history of intravenous drugs, and elevated serum lactate dehydrogenase (LDH) [119]. In the same vein, GICAT (Gruppo Italiano Cooperativo AIDS e Tumori) and GELA (Groupe d’Etude des Lymphomes de l’Adulte) groups performed an index combining three independent risk factors: ECOG (Eastern Cooperative Oncology Group), performance status of 2 or more, prior AIDS defining illness, and CD4^+^-cell count <100/μL [120]. 

In the pre-cART era, outcomes were poorer and highly influenced by hematological toxicity (complete remission rates of about 50% and 5-year OS of about 28–48%), with a huge difference compared to HIV-negative patients [12]. In the cART era, the paradigm of therapy has significantly changed in PWH with lymphomas. In addition to better control of viral load, better management of the chemotherapy regimens and better supportive care is carried out, and survival is now reaching that of patients with non-related-HIV NHL [12]. Therefore, currently HIV-related DLBCL prognosis is determined by lymphoma features: international prognostic score (IPI), revised-IPI [121,122], or age-adjusted IPI, rather than HIV-specific factors [17]. Some authors have focused on the need for refinement of the aa-IPI score in patients with HIV-related NHL, due to the HIV infection being a competing risk that may influence prognosis. In 2014, Barta et al. developed a new prognostic index called ARL (AIDS-related lymphoma)-IPI, which consists of three components: aa-IPI, number of involved extranodal sites, and an HIV score that incorporates baseline CD4^+^ count, HIV viral load, and prior history of AIDS [123]. 

Despite the prognosis predicting power of the IPI being proved in HIV-related DLBCL [123,124,125,126,127], new scores with a more conscious predictive ability have been developed (National Cancer Comprehensive Network IPI (NCCN-IPI) [128,129], GELTAMO-IPI [130], and another new score which includes data from peripheral blood count [131]), including new variables such as beta2-microglobulin and/or lymphocyte and monocyte count, is still not validated in the HIV-related DLBCL. 

## 5. Future Directions

There are still some unmet questions to be addressed about DLBCL affecting PWH. Regarding etiopathogenesis, further research is needed to establish the real differences between DLCBL in PWH and the general population. Some studies already point out that DLCBL in both settings have important similarities regarding the phenotypic and genetic features [42], but they may have some differences as well. The role of EBV should be further investigated by applying techniques that could unveil unknown viral mechanisms of action, with special focus on the hit and run hypothesis.

The different prognostic scores, designed to predict the prognosis of DLBCL such as IPI, age-adjusted IPI, NCCN, and GELTAMO-IPI should be validated in the HIV setting. These studies could provide more arguments in favor of the fact that the prognosis of HIV-DLBCL nowadays depends more on lymphoma variables than on those of HIV infection. The results of these studies would presumably provide reasons to not exclude PWH with DLCBL from clinical trials anymore. This would be a big step toward the goal of giving PWH the same treatment options for DLBCL and other lymphomas as the general population.

## 6. Conclusions

PWH have an increased risk of hematologic cancers, and lymphoma is a leading cause of morbidity and mortality in these patients. DLBCL is the most common NHL subtype affecting PWH. There are two morphological variants of HIV-related DLBCL, centroblastic variant and immunoblastic variant, the latter occurring with more frequency in patients with a higher grade of immunosuppression. The widespread use of cART led to a decreased incidence of DLBCL, especially of the immunoblastic variant. Although the information on HIV-related DLBCL pathogenesis is scarce, HIV is not known to have direct oncogenic effects, but HIV infection causes several indirect effects contributing to lymphomagenesis. With the introduction of cART, PWH presented an improved immune function, and the incorporation of additional supportive care measures (such as infection prophylaxis or the use of hematopoietic growth factors) allowed the use of conventional standard-dose chemotherapy regimens for initial treatment and as treatment of relapses. Although concomitant administration of chemotherapy with cART may be challenging due to drug–drug interactions and overlapping toxicity, patients with HIV-related DLBCL should start or maintain cART concomitantly with the chemotherapy regimen, since this concomitant use has demonstrated an improvement of the complete response rate. In the cART era, lymphomas occurred in a low proportion of PWH, suggesting that the persistent antigenic stimulation and HIV-released proteins are not enough to trigger lymphomagenesis. The role of the HIV in microenvironment regulation and favoring B-cell expansion is still misunderstood and is a current research line. Although survival is now on the way to reaching that of patients with non-related-HIV DLBCL, more aggressive clinical features are still present in DLBCL in PWH. The inclusion of PWH treated with cART in clinical trials and the implementation of novel therapies in this group of patients are still a challenge. Interdisciplinary collaboration between hematologists and HIV specialists is crucial for the optimal treatment of both conditions, minimizing risk of adverse outcomes in the patient. 

## Figures and Tables

**Table 1:** Distribution of lymphoma subtypes in people with HIV through 3 decades. Data from Center for AIDS Research (CFAR) Network of Integrated Clinical Systems (CNICS) USA cohort of 476 patients [5].

	1996–2000 CNICS (*n* = 132)	2001–2005 CNICS (*n* = 201)	2006–2010 CNICS (*n* = 143)	Trend
DLBCL * (%)	43.9	45.8	35.7	↓
BL * (%)	7.6	10.9	16.8	↑
PCNSL * (%)	14.4	10.4	9.8	↓
HL * (%)	15.2	15.4	19.6	↑
Others (%)	18.9	17.4	18.2	=

* DLBCL: diffuse large B-cell lymphoma; BL: Burkitt lymphoma; PCNSL: primary central nervous system lymphoma; HL: Hodgkin lymphoma.

**Table 2:** DLBCL morphological variants in PWH: pathologic and immunophenotype markers, virologic co-infection, and genetic features. Adapted from Carbone et al. [12,17].

	CD20	BCL6	IRF4/ MUM1	CD138	Other Cell Markers	EBV Infection	HHV-8 Infection	Genetic Features
DLBCL, immunoblastic-plasmacytoid	+ (may be lost)	-	+	+	CD10, CD30, CD5	Positive (90–100%)	Negative (some positive)	*TP53* point mutation, *MYC* rearrangement, *RAS* point mutation, *BCL2* and *BCL6* rearrangements
DLBCL, centroblastic	+	+	-	-	CD10, CD5	Positive (25–30%)	Negative	

**Table 3:** Pivotal clinical trials with most commonly used chemotherapy regimens in HIV-related DLBCL. Extended from Carbone et al. [17] and the references specified in the table.

	CHOP/R-CHOP/DR-COP				CDE/R-CDE		R-EPOCH/SC-EPOCH-RR	
	Boué et al., 2003 [74]	Kaplan et al., 2005 [73]	Ribera et al., 2008, 2012 [75,76]	Levine et al., 2013 [78]	Sparano et al., 2004 [67]	Spina et al., 2005 [71]	Sparano et al., 2010 [64]	Dunleavy et al., 2010 [41]
No. of patients	61	150	81	40	98	74	106	33
Study design	Phase II R-CHOP	Phase III CHOP vs. R-CHOP	Phase II R-CHOP	Phase II DR-COP	Phase II CDE	Pooled results from 3 phase II trials R-CDE	Phase II R-EPOCH vs. EPOCH-R	Phase II SC-EPOCH-RR
DLBCL histology, N (%)	44 (72)	120 (80)	81 (100)	39 (98)	76 (78)	53 (72)	79 (75)	33 (100)
CD4**^+^** μL, median	172	133	158	114	160	161	181 vs. 194	208
aa-IPI ≥ 2, N (%)	29 (48)	70 (47)	55 (67)	11 (27)	62 (67)	42 (57)	70 (66)	25 (76)
Outcome, %								
CR * rate	77	47 vs. 58	69	47	45	70	73 vs. 55	91
PFS *	69 (2-year)	9.5 vs. 11.3 months	-	52 (2-year)	36 (2-year)	59 (2-year)	66 vs. 63 (2-year)	84 (5-year)
OS *	75 (2-year)	28 vs. 35 months	56 (3-year)	62 (2-year)	43 (2-year)	64 (2-year)	70 vs. 67 (2-year)	68 (5-year)
Infectious deaths (%)	2	2 vs. 14	7	0	Unknown	7	10 vs. 7	0

* CR: complete response, PFR: progression-free survival, OS: overall survival.
